# The Usefulness of Serum Biomarkers in the Early Stages of Diabetic Retinopathy: Results of the EUROCONDOR Clinical Trial

**DOI:** 10.3390/jcm9041233

**Published:** 2020-04-24

**Authors:** Cristina Hernández, Massimo Porta, Francesco Bandello, Jakob Grauslund, Simon P. Harding, Stephen J. Aldington, Catherine Egan, Ulrik Frydkjaer-Olsen, José García-Arumí, Jonathan Gibson, Gabriele E. Lang, Rosangela Lattanzio, Pascale Massin, Edoardo Midena, Berta Ponsati, Luísa Ribeiro, Peter Scanlon, José Cunha-Vaz, Rafael Simó

**Affiliations:** 1 Diabetes and Metabolism Research Unit and CIBERDEM, Vall d’Hebron Research Institute, 08035 Barcelona, Spain; rafael.simo@vhir.org; 2 Department of Medical Sciences, University of Turin, 10124 Turin, Italy; massimo.porta@unito.it; 3 Department of Ophthalmology, Scientific Institute San Raffaele, University Vita-Salute, 20132 Milano, Italy; francesco.bandello@uniud.it (F.B.); lattanzio.rosangela@hsr.it (R.L.); 4 Research Unit of Ophthalmology, Department of Clinical Research, University of Southern Denmark, 5230 Odense, Denmark; Jakob.Grauslund@rsyd.dk (J.G.); ulrik.frydkjaer-olsen@rsyd.dk (U.F.-O.); 5 Department of Eye & Vision Science, Institute of Ageing and Chronic Disease, University of Liverpool, and St. Pauls’ Eye Unit. Liverpool University Hospitals, members of Liverpool Health Partners, Liverpool L69 7ZX, UK; S.P.Harding@liverpool.ac.uk; 6 Gloucestershire Hospitals National Health Service Foundation Trust, Cheltenham GL53 7AG, UK; Steve.Aldington@glos.nhs.uk (S.J.A.); p.scanlon@nhs.net (P.S.); 7 Moorfields Eye Hospital National Health Service Foundation Trust, Institute of Ophthalmology/University College London, London EC1V 2PD, UK; catherine.egan@moorfields.nhs.uk; 8 Department of Ophthalmology, Vall d’Hebron University Hospital, 08035 Barcelona, Spain; jgarcia.arumi@gmail.com; 9 Department of Vision Sciences, Aston University, Birmingham B4 7ET, UK; j.m.gibson@aston.ac.uk; 10 Department of Ophthalmology, University of Ulm, 89081 Ulm, Germany; gabriele.lang@uniklinik-ulm.de; 11 Department of Ophthalmology, Lariboisière Hospital, 75004 Paris, France; pascale.massin@lrb.aphp.fr; 12 Department of Ophthalmology, University of Padova, 35122 Padova, Italy; edoardo.midena@unipd.it; 13 BCN Peptides, 08777 Barcelona, Spain; bponsati@bcnpeptides.com; 14 Association for Innovation and Biomedical Research on Light and Image (AIBILI), 3000-548 Coimbra, Portugal; lr@aibili.pt (L.R.); cunhavaz@aibili.pt (J.C.-V.)

**Keywords:** diabetic retinopathy, retinal neurodegeneration, serum biomarkers, laminin, carboxy methyl lysine, asymmetric dimethylarginine

## Abstract

The main aim of this study was to evaluate the ability of serum biomarkers to predict the worsening of retinal neurodysfunction in subjects with type 2 diabetes. For this purpose, we measured selected molecules (N-epsilon-carboxy methyl lysine (CML), laminin P1 (Lam-P1), and asymmetric dimethylarginine (ADMA)) in the serum of 341 participants of the EUROCONDOR study at baseline, 24, and 48 weeks. Retinal neurodysfunction was assessed by measuring implicit time (IT) using multifocal electroretinography, and structural changes were examined by spectral domain–optical coherence tomography. The values of IT at baseline were directly correlated with baseline serum concentrations of CML (*r* = 0.135, *p* = 0.013). Furthermore, in the placebo group, increase in CML concentration throughout follow-up correlated with the IT (*r* = 0.20; *p* = 0.03). Baseline serum levels of CML also correlated with macular retinal thickness (RT) (*r* = 0.231; *p* < 0.001). Baseline Lam-P1 levels correlated with the increase of the RT at the end of follow-up in the placebo group (*r* = 0.22; *p* = 0.016). We provide evidence that CML may be a biomarker of both retinal neurodysfunction and RT, whereas Lam-P1 was associated with RT only. Therefore, circulating levels of these molecules could provide a complementary tool for monitoring the early changes of diabetic retinopathy (DR).

## 1. Introduction

Diabetic retinopathy (DR) is the most frequent complication of diabetes and the main cause of visual impairment in working-age adults in developed countries [1,2]. Current treatments for DR, such as laser photocoagulation and intravitreal injections of corticosteroids or anti-vascular endothelial growth factor (VEGF) agents, are applicable only at advanced stages of the disease, and are associated with significant adverse effects [3,4]. Fenofibrate and calcium dobesilate have been used as oral agents in the early stages of DR, but they are not included in current clinical guidelines [5]. Therefore, in the early stages of DR, the only therapeutic strategy that physicians can offer is a tight control of the main modifiable risk factors, such as glycemia and blood pressure. However, clinical studies in patients with diabetes reveal a substantial variation in the onset and severity of DR, thus indicating that genetic factors may influence the susceptibility of DR development and progression [6,7,8].

Circulating biomarkers could be useful for detecting early retinal disease before structural changes can be clearly seen using current imaging techniques. In addition, they may help to identify patients with diabetes that is more prone to progressive worsening, in whom intensified therapy could be prioritized, and to monitor the effectiveness of new drugs for DR before its advanced stages have developed. Research on biomarkers has been mainly based on the pathogenic mechanism involved in the development of DR, such as advanced glycation end products (AGEs), endothelial dysfunction, inflammation, and basement membrane and extracellular matrix turnover [9,10].

In recent years, the concept that neurodegeneration is an early event in the pathogenesis of DR has emerged, and pre-clinical evidence suggests that neuroprotection can be a therapeutic target [11,12]. In addition, several phenotypes based on retinal examination have been identified [13,14]. These findings have renewed the interest of circulating biomarkers as an important tool to complement the information provided by retinal assessments.

The EUROCONDOR study included a phase II–III randomized, controlled clinical trial aimed at evaluating the effects of topical neuroprotection in arresting or preventing early retinal neurodegeneration in DR [14,15]. The selected neuroprotective agents appear useful in preventing the worsening of preexisting retinal neurodysfunction [15]. This finding points to screening for retinal neurodysfunction as a critical issue, in order to identify a subset of patients in whom neuroprotective treatment might be of benefit. In this study, we wanted to test whether some circulating molecules can act as serum biomarkers in predicting either the worsening of retinal neurodysfunction, as assessed by multifocal electroretinography (mfERG), or structural changes, as assessed by a combination of spectral domain–optical coherence tomography (SD-OCT) and seven-field fundus photographs.

The selected molecules were based on three essential pathogenic factors involved in the very early stages of DR: basement membrane thickening [16,17], the accumulation of advanced glycation end-products (AGEs) [18,19], and oxidative stress [20,21].

As a biomarker of the increased thickening and turnover of the basement membrane that exists in diabetes, we selected laminin P1 (the largest pepsin resistant fragment of laminin), which is the main non-collagenous component of the basement membrane. Basement membranes of retinal vessels in diabetic rats contain increased amounts of laminin, as early as 8 weeks after the induction of diabetes, indicating increased expression of matrix components [22]. In addition, in a prospective study we found that circulating laminin-P1 was an early marker of the presence of DR, as well as a marker of its severity [23].

The selected molecule among AGEs was N-epsilon-carboxy methyl lysine (CML), which is found in the normal retina and with concentration greatly increased in the neuroglial and vascular components of the retina of patients with diabetes [24]. There is mounting evidence on the deleterious effects of AGEs in the early stages of DR [25,26,27], and their role in neurodegeneration has been comprehensively reviewed [28]. In addition, CML has been found to be elevated in the serum of patients with diabetes, and to an even greater extent in those with microvascular complications [9]. Furthermore, skin collagen CML levels, measured in human skin punch biopsy samples, predicted the progression of DR in a prospective study [29].

For testing oxidative stress—and more specifically, endothelium-derived oxidative stress—we have used serum asymmetric dimethylarginine (ADMA). Endothelial dysfunction and impaired ocular hemodynamics underlying DR development are associated with decreased nitric oxide (NO) synthase activity and NO bioavailability, thus resulting in vasoconstriction and increased reactive oxygen species (ROS) [30]. Serum asymmetric dimethylarginine (ADMA) is involved in the NO pathway, and serum levels of ADMA have been found to be elevated in patients with diabetes and DR [31,32]. Increased oxidative stress contributes to elevated ADMA. In turn, increased serum ADMA concentration is associated with increased vascular oxidative stress, as demonstrated by the upregulation of circulating markers of oxidative stress [30].

## 2. Experimental Section

EUROCONDOR [NCT01726075] was a 96 week, European multicenter, prospective, interventional, phase II–III, randomized controlled clinical trial aimed at evaluating the effect of topical (eye drops) neuroprotective agents to arrest or prevent early retinal neurodegeneration in DR [14,15]. Briefly, 449 patients were recruited in 11 European centers and randomized 1:1:1 to topical treatment twice daily with a placebo, brimonidine tartrate 0.2%, or somatostatin 0.1%.

The inclusion criteria were type 2 diabetes with no, minimal, or mild DR (Early Treatment Diabetic Retinopathy Study (ETDRS) levels: 10, 20, or 35); known duration of diabetes at least 5 years; and age between 45 and 75 years. Relevant to this report, microvascular changes were assessed by using standard seven-field color fundus photography. Retinal neurodysfunction was assessed by mfERG, implicit time (IT) being the primary end-point. Structural changes were assessed by spectral domain–optical coherence tomography (SD-OCT). One eye per patient was included in the study. If both eyes met the inclusion criteria, one eye was chosen randomly.

The study was approved and funded by the European Commission Seventh Framework Program (grant agreement no. FP7–278040). At all centers, the study was conducted in accordance with the tenets of the Declaration of Helsinki, with approvals of the local scientific ethnical committees; written informed consent was obtained from all patients.

### 2.1. Measurement of Biomarkers

Blood samples for potential biomarkers were taken at the screening visit (*n* = 449) and at weeks 24 and 48. The molecules were analyzed in patients that completed the 2 years of follow-up (*n* = 341). Serum circulating biomarkers were measured using the following enzyme-linked immunosorbent assay (ELISA) kits: Lam P1 fragment (cat. MBS9310966, MyBiosource, San Diego, California, United States), CML (cat. STA-816, Cell Biolabs Inc, San Diego, California, United States), and ADMA (cat. CEB301Ge, Cloud-Clone Corp., Wuhan, Hubei, China). Details of the ELISA kits are displayed in Table 1.

### 2.2. Statistical Analyses

Because of their skewed distribution CML, laminin P1 (Lam-P1), and asymmetric dimethylarginine (ADMA) were log-transformed. For continuous variables, paired and unpaired Student’s *t*-tests were used. Results are expressed as the mean ± SD (standard deviation) or as the median (range). To evaluate correlations, the Spearman’s correlation test was performed. Received operated curves (ROC) curves and the Chi-squared test for the area under the ROC curve (AUC) comparison was performed. All *p* values are based on a two-sided test of statistical significance. Significance was accepted at the level of *p* < 0.05. Statistical analyses were performed with the Stata statistical package.

## 3. Results

The main clinical and laboratory parameters of patients included in the study at baseline, according to treatment, are shown in Table 2.

### 3.1. Usefulness of Serum Biomarkers for Monitoring Neurodysfunction

**Figure 1:**
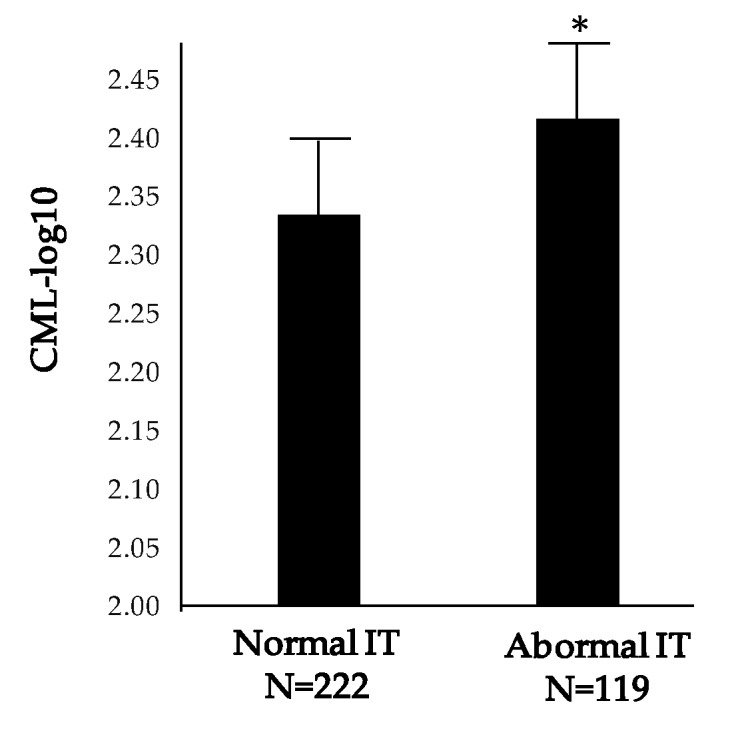
Comparison of baseline serum levels of N-epsilon-carboxy methyl lysine (CML; log-transformed) between patients with normal and abnormal implicit time (IT; IT 37.43 milliseconds). * *p* = 0.009. AU: arbitray units.

The values of IT at baseline were directly correlated with baseline serum concentrations of CML (*r* = 0.135, *p* = 0.013). Thus, serum levels of CML were significantly higher in subjects with elevated IT in comparison with those with an IT within the normal range, according to the normative database previously developed by the EUROCONDOR Consortium [33] (Figure 1). As mentioned above, we did not find any correlation between CML levels and HbA1c, which, in turn was unrelated to IT.

**Figure 2:**
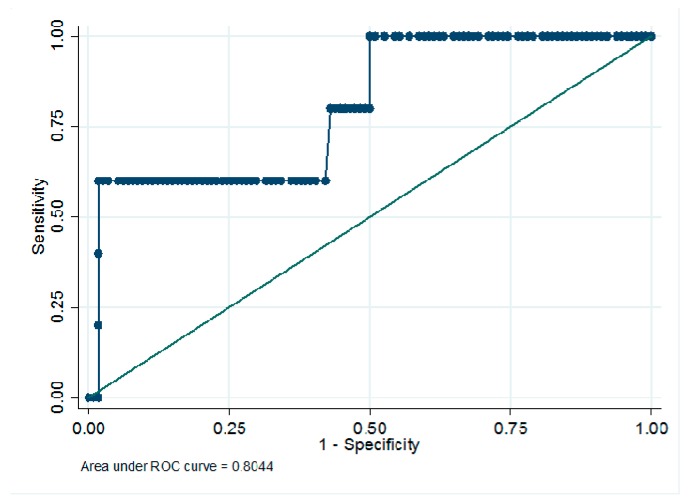
Prediction model of worsening of IT (increase of >2 SDs at the end of follow-up) based on the increase of serum CML levels during the first year of follow-up. AU: 0.804 (95% CI: 0.585–1). AU: arbitray units.

The increase in CML concentration throughout the follow-up was correlated to the worsening of IT in the placebo group (*r* = 0.20; *p* = 0.03). However, this direct correlation was not observed in the groups treated with brimonidine or somatostatin. The AU of CML in predicting the increase in ≥2 SDs of IT in those patients treated with the placebo was 0.804 (95% CI: 0.585–1) (Figure 2).

We did not observe any relationship between either LamP-1 or ADMA and IT.

### 3.2. Usefulness of Serum Biomarkers to Monitoring Structural Changes

Baseline serum levels of CML correlated with macular retinal thickness (RT) (*r* = 0.231; *p* < 0.001). In fact, diabetic patients with subclinical diabetic macular edema, as defined by The Diabetic Retinopathy Clinical Research Network (DRCR.net) [34], presented higher levels of CML than those with normal RT (CML log: 2.42 ± 0.22 vs. 2.32 ± 0.28; *p* = 0.003). In addition, a correlation between CML and ganglion cell layer–inner plexiform layer (GCL-IPL) thickness was observed (*r* = 0.156; *p* = 0.030, respectively).

**Figure 3:**
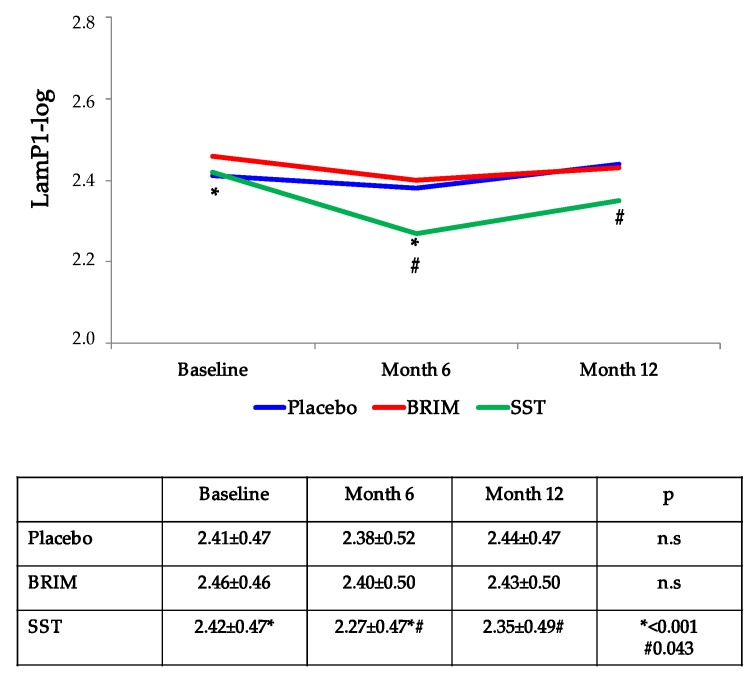
Evolution of Lam-P1 levels (log transformed) in the three therapeutic arms. Lam-P1 levels significantly decrease in type 2 diabetes mellitus patients treated with somatostatin. Lam-P1: laminin P1; BRIM: brimonidine; SST: somatostatin. * *p* < 0.001 between Baseline and Month 6. # *p* = 0.043 between Month 6 and Month 12.

A correlation between baseline Lam-P1 levels and GCL-IPL thickness was observed (*r* = 0.114; *p* = 0.036). In addition, baseline Lam-P1 levels correlated with the increase of the RT at the end of follow-up in the placebo group (r = 0.22; *p* = 0.016), but not in the groups treated with the neuroprotective drugs (brimonidine or somatostatin). Interestingly, a significant decrease in the serum Lam-P1 was observed in the group treated with somatostatin (SST) at 6 and 12 months. This effect was not seen in the placebo and brimonidine groups (Figure 3).

We did not find any relationship between the serum levels of ADMA and any of the structural parameters measured by SD-OCT.

### 3.3. Usefulness of Serum Biomarkers in Monitoring Early Microvascular Changes

We did not find any relationship between the severity of DR (ETDRS level) and the serum levels of the selected molecules (CML, Lam-P1, and ADMA) (Table 3). Their possible relationship to progression in the ETDRS scale could not be analyzed, because fewer than 10 patients increased by at least one step in ETDRS scale during the 2 years of follow-up.

## 4. Discussion

This is the first study aimed at evaluating the usefulness of circulating biomarkers in the early stages of DR in a large and well-characterized cohort of patients with type 2 diabetes. We provide evidence that CML is a biomarker for both neurodysfunction and RT, whereas Lam-P1 may represent a biomarker for RT only. We did not find any relationship between the serum levels of ADMA and either neurodysfunction or structural changes in the retina.

Neurodysfunction is an early abnormality in the natural history of DR, detected even before the structural changes of the neurodegenerative process [11,12]. In the present study, we found a direct and significant correlation between baseline serum levels of CML and retinal neurodysfunction, assessed by the IT in patients with type 2 diabetes without DR or with only mild DR. In addition, the increase in CML concentrations throughout the study was related to impairment of IT in the placebo group, which represents the natural history of DR evolution, whereas such a relationship was no longer observed in the arms treated with neuroprotective drugs. This is an important finding, because both brimonidine and somatostatin have appeared able to ameliorate the increase of IT, thus preventing further worsening of neurodysfunction [15]. Hence, as CML ran in parallel to the IT throughout the study, it could be a useful marker for monitoring the effects of neuroprotective drugs, at least in terms of neurodysfunction.

Notably, no relationship between HbA1c and CML was observed, and this finding emphasizes the limited value of isolated values of HbA1c in capturing all the glycation process that occurs in diabetes.

CML, the most abundant among circulating AGEs, was elevated in the serum of patients with diabetes, and to an even greater extent in those with microvascular complications [35,36,37,38,39], including those patients with DR [40]. However, to the best of our knowledge, this is the first evidence that CML may be useful in detecting retinal neurodysfunction and predicting its worsening. In this regard, it should be noted that AGEs have been involved in the pathogenesis of neurodegenerative diseases like Alzheimer’s [41,42], whereas a relationship with retinal neurodysfunction has not been previously examined.

In addition, we found that serum levels of CML significantly correlated with retinal thickness at baseline, and in fact, those patients with diabetic macular subedema (23% of patients of the EUROCONDOR cohort) presented CML values significantly higher than patients with normal RT. This finding could be important to complement the SD-OCT information, thus improving the phenotyping of DR. In addition, the serum levels of CML could be useful in monitoring the effects of new drugs for treating early stages of diabetic macula edema.

Baseline levels of Lam-P1 correlated with GCL-IPL thickness, and were directly related to the increase in RT at the end of the follow-up in the placebo group. These findings support previous reports indicating that Lam-P1 may be a biomarker of basement membrane thickness and its turnover in diabetes-induced microvascular disease [23]. Interestingly, in the present study, Lam-P1 levels decreased in patients treated with SST, thus suggesting a direct effect of SST on this essential component of the basement membrane. This was certainly intriguing, because SST was topically administered, and retinal microcirculation represents only a minor part of the total number of blood vessels in the body. In this regard, we previously reported that panretinal photocoagulation significantly reduced serum Lam-P1 levels in patients with diabetes [43]. Altogether, these findings suggest that the contribution of retinal basement membrane turnover to circulating Lam-P1 is not negligible.

Growing evidence suggests that retinal neurodegeneration is an early event in the pathogenesis of DR, and could contribute to further development of microvascular abnormalities [11,12]. However, in the EUROCONDOR study, we found that this may not be a universal pattern, and that in a significant proportion of patients the development of microvascular disease is not preceded by any neurodegenerative abnormalities [14,15]. In this regard, our results suggest that CML and Lam-P1 could represent useful biomarkers to identify and monitor these different phenotypes. It should be noted that mfERG is a cumbersome and time-consuming procedure requiring specialized personnel, and is mainly reserved for clinical trials. The measurement of biomarkers would be simpler and cheaper than mfERG. Therefore, in those patients in whom neurodysfunction has been identified, the assessment of the proposed biomarkers, particularly CML, could be useful in monitoring the natural history of the disease and the effect of treatment. In addition, this strategy could allow one to skip mfERG during follow-up or replace it with another, simpler examination, such as microperimetry. Furthermore, the measurements of biomarkers could give us complementary information about the impairment of the neurovascular unit. Nevertheless, although promising, our results should be validated in other cohorts.

It must be noted that serum concentrations of the biomarkers measured could be influenced by other microangiopathic complications, with particular reference to the kidney function. However, in the EUROCONDOR clinical trial, patients with renal failure (creatinine > 123.76 µmol/L) were excluded, and the results remain similar when adjusted by presence of microalbuminuria. The main limitation of our study is the low progression rate of microvascular disease, due to the short follow-up (2 years). This limiting factor precludes any conclusion being drawn on the usefulness of the selected biomarkers in terms of microvascular progression. Therefore, further clinical research with a longer follow-up is needed.

In conclusion, we found that CML and LamP1 could help us identify subjects with type 2 diabetes with the early stages of DR. The changes of these biomarkers could be a complementary tool for the assessment of neurodysfunction and retinal thickness in subjects with type 2 diabetes, and could also provide useful information when monitoring the effectiveness of treatments in the early stages of DR.

## Figures and Tables

**Table 1:** Sensitivity and intra- and inter-assay coefficients of the measured circulating biomarkers.

	Sensitivity	Intra-assay coefficient	Inter-assay coefficient
CML	2.25 ng/mL	<10%	<12%
Lam-P1	5.00 ng/mL	<8%	<12%
ADMA	4.34 ng/mL	<8%	<12%

CML: N-epsilon-carboxy methyl lysine; Lam-P1: Laminin P1; ADMA: asymmetric dimethylarginine;

**Table 2:** Baseline characteristics of type 2 diabetes mellitus patients included in the analysis of efficacy.

	Placebo	Brimonidine	Somatostatin
	*n* = 123	*n* = 97	*n* = 120
Age (years)	62.4 ± 7.1	63.7 ± 6.0	62.6 ± 6.6
Gender (% males)	66.1	66.0	65.0
BMI (Kg/m^2^)	30.8 ± 5.6	30.8 ± 5.3	31.1 ± 5.4
Diabetes duration (years)	11.6 ± 5.8	11.1 ± 5.5	11.4 ± 5.5
Diabetes treatment (%)			
Diet	4.8	2.1	4.2
Oral agents	65.3	76.3	73.3
Oral agents + insulin	24.2	21.6	20.8
Insulin	5.6	0.0	1.7
HbA1C (%)	7.21 ± 0.97	7.22 ± 1.09	7.11 ± 0.92
Hypertension (%)	71.0	73.2	71.7
Dyslipidemia (%)	69.4	67.0	67.5
Microalbuminuria (%)	19.3	22.7	19.1
Cardiovascular disease (%)	19.4	14.4	21.7
ETDRS 10/20-35(%)	42.7/57.3	38.1/61.9	43.3/56.7
BCVA letter score	85.9 ± 5.2	86.1 ± 5.2	85.7 ± 4.6

At baseline, the serum levels of CML and LamP-1 were not associated with age, gender, BMI, duration of diabetes, fasting plasma glucose, or hemoglobin A1c (HbA1c). A direct correlation was observed between ADMA and HbA1c (*r* = 0.13; *p* = 0.016); however, as occurred with CML and Lam-P1, we did not detect any relationship between ADMA and age, gender, BMI, or duration of diabetes. BMI: body mass index; ETDRS: Early Treatment Diabetic Retinopathy Study; BCVA: best corrected visual acuity.

**Table 3:** Baseline serum levels of biomarkers compared to severity of DR (ETDRS classification).

	ETDRS 10	ETDRS 20-35	
	*n* = 140	*n* = 201	*p*
CML (log-transformed)	2.35 ± 0.28	2.36 ± 0.26	n.s.
Lam-P1 (log-transformed)	2.46 ± 0.47	2.41 ± 0.47	n.s.
ADMA (log-transformed)	1.91 ± 0.27	1.90 ± 0.30	n.s.

n.s. Not significant.
